# *‘Le plus important est invisible*’: congenital pericardial defect with structural, electrophysiological, and haemodynamic alterations induced by postural changes—a case report

**DOI:** 10.1093/ehjcr/ytaf200

**Published:** 2025-04-21

**Authors:** Manami Shingu, Kensuke Matsumoto, Tetsu Yamazaki, Satoru Kawasaki, Hogara Nishisaki

**Affiliations:** Division of Cardiovascular Medicine, Department of Internal Medicine, Hyogo Prefectural Tamba Medical Center, 2002-7, Isou, Hikami-cho, Tamba-shi, Hyogo, Tamba 669-3495, Japan; Division of Cardiovascular Medicine, Department of Internal Medicine, Hyogo Prefectural Tamba Medical Center, 2002-7, Isou, Hikami-cho, Tamba-shi, Hyogo, Tamba 669-3495, Japan; Division of Cardiovascular Medicine, Department of Internal Medicine, Hyogo Prefectural Tamba Medical Center, 2002-7, Isou, Hikami-cho, Tamba-shi, Hyogo, Tamba 669-3495, Japan; Division of Cardiovascular Medicine, Department of Internal Medicine, Hyogo Prefectural Tamba Medical Center, 2002-7, Isou, Hikami-cho, Tamba-shi, Hyogo, Tamba 669-3495, Japan; Division of Cardiovascular Medicine, Department of Internal Medicine, Hyogo Prefectural Tamba Medical Center, 2002-7, Isou, Hikami-cho, Tamba-shi, Hyogo, Tamba 669-3495, Japan

**Keywords:** Congenital pericardial defect, postural change, hanging down, fixation of the heart, multimodality imaging, case report

## Abstract

**Background:**

The principal roles of the pericardium include protection from microorganisms, prevention of cardiac friction, and restriction of unlimited dilation of the heart. In the case of a congenital pericardial defect in which structural, electrophysiological, and haemodynamic abnormalities manifested during postural changes, we propose another indispensable pericardial function of cardiac central anchorage.

**Case summary:**

A 29-year-old man with atypical chest pain was referred to our hospital. Electrocardiography revealed fluctuations in the QRS transitional zone, electrical axis, and atrial polarity with body posture. Echocardiography revealed a far dorsally displaced heart, paradoxical motion of the interventricular septum (IVS), and hyperdynamic motion of the posterior wall in the left lateral decubitus position, which normalized to the right lateral decubitus position, along with significant haemodynamic alterations. Multidetector-row computed tomography revealed a complete absence of the left pericardium.

**Discussion:**

When pericardial fixation is impaired, the heart falls dorsally owing to gravity in the left lateral decubitus position, resulting in right ventricular overstretch and enlargement, which, in turn, results in compression of the left ventricle via a leftward shift of the IVS and a consequent reduction in cardiac output. Moreover, the energy generated by the myocardium, which should normally be concentrated only on blood ejection, would be distributed between the energy used for ejection and that used for the leap-up movement of the heart, reducing its energy efficiency. Through detailed observation of the congenital pericardial defect, haemodynamic insights into the important functions of the pericardium, which were not visible through static observation, were obtained.

**Learning points**

- In pericardial defects, impaired fixation and absence of restraint of the pericardium can result in haemodynamic disadvantages depending on body posture.
- Firm anchorage of the heart may be another important function of the pericardium, which is not visible through static observations.
- A comprehensive patient history, including an assessment of trepopnoea, meticulous evaluation of postural changes during physical examination, and detailed imaging analysis, plays a crucial role in the accurate diagnosis of this congenital condition.

## Introduction

The principal role of the pericardium is to exert physical functions, including protection of the heart from extrinsic pathogenic microorganisms^1^ and prevention of friction with adjacent structures.^2^ It prevents unlimited dilation of the heart against an abrupt increase in cardiac preload and suppresses excessive cardiac motion during the cardiac cycle.^3,4^ Furthermore, it anchors the heart to the centre of the mediastinum, which might be another non-negligible function of the pericardium.^5^

Herein, we present detailed structural, electrophysiological, and haemodynamic observations on the essential function of the pericardium, particularly its role in the central fixation of the heart, through a case of congenital pericardial defect.

## Summary figure

**Figure:**
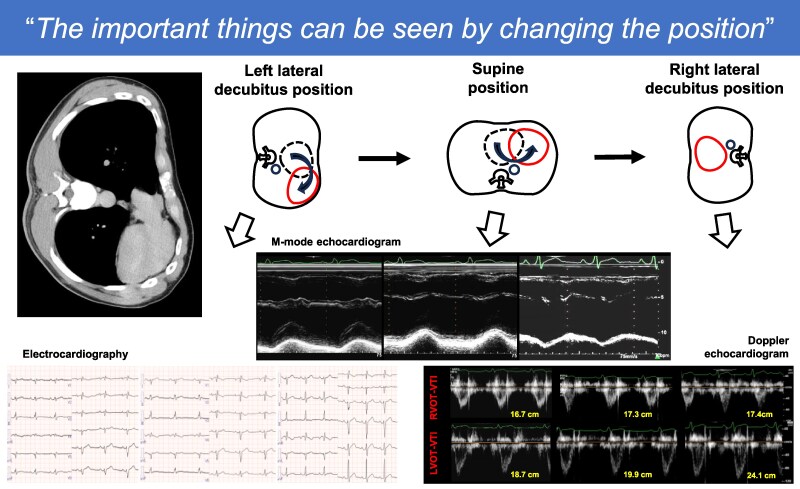


## Case presentation

A 29-year-old man with atypical chest pain was referred to our hospital. His medical history was only remarkable for a right-sided aortic arch and aberrant left subclavian artery, which were incidentally diagnosed during childhood. Unexplained ‘rotation’ and ‘malposition’ of the heart were pointed out at a tertiary children’s hospital. Subsequently, he experienced intermittent chest pain and visited several medical facilities, but no major abnormalities were identified. He also had previously found it easier to remain in the right-sided lying position (i.e. trepopnoea). He had no known family history of cardiovascular diseases.

**Figure 1:**
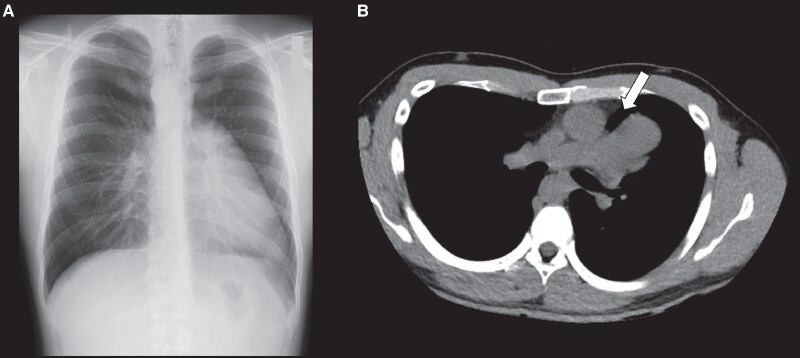
Chest radiography and computed tomography of a 29-year-old man. Chest radiograph showing a leftward shift of the cardiac silhouette with no tracheal deviation, prominent pulmonary arteries, and interposed lung tissue, causing a radiolucent zone between the heart and the diaphragm (*A*). Computed tomography showing interposition of the pulmonary tissue between the aorta and main pulmonary artery (white arrow), indicating the presence of a congenital pericardial defect (*B*).

**Figure 2:**
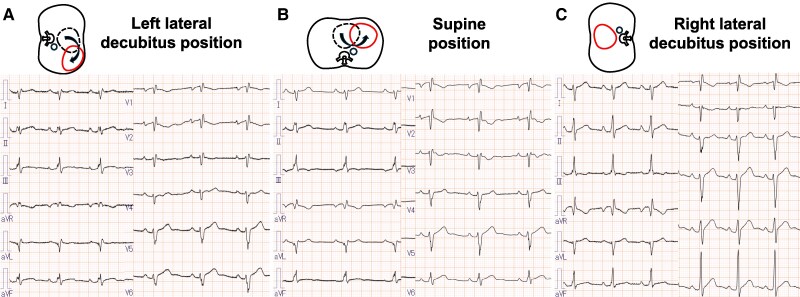
Electrocardiography of a 29-year-old man in various body positions. In the left lateral decubitus position (*A*), there is no transition zone with a QRS axis of 98° and the P wave of the V1 lead is mostly negative in terms of voltage. In the supine position (*B*), a transition zone was observed between V5 and V6, with a QRS axis of 111°. In the right lateral decubitus position (*C*), the transition zone moved further between V4 and V5, with a QRS axis of 132° and the polarity of the P wave of V1 changed to positive.

**Figure 3:**
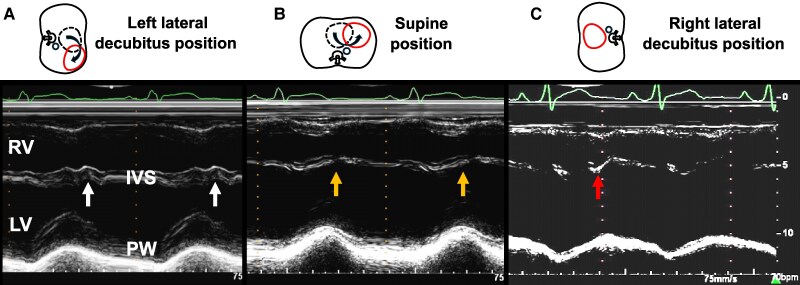
In the left lateral decubitus position (*A*), hyperdynamic motion of the left ventricular (LV) posterior wall and paradoxical motion of the interventricular septum (IVS, arrows) are clearly observed. Enlargement of the right ventricular (RV) cavity was also observed. In the supine position (*B*), both hyperdynamic posterior wall motion and paradoxical IVS motion (arrows) were alleviated. Furthermore, in the right lateral decubitus position (*C*), the hyperdynamic motion of the posterior wall, paradoxical motion of the IVS (arrow), and RV dilation disappeared.

**Figure 4:**
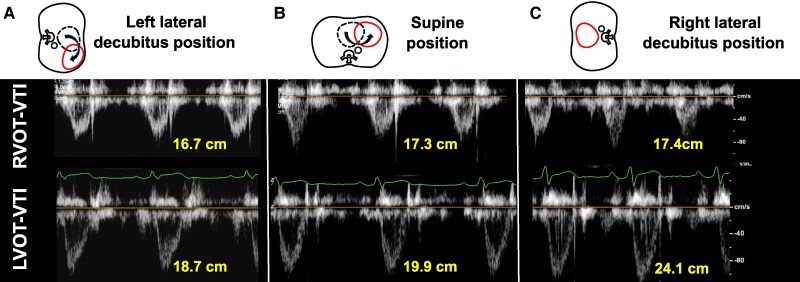
Doppler echocardiogram from RV and LV outflow tract of a 29-year-old man in various body positions. Compared with the right lateral decubitus position (*C*, right panel), the RV and LV outflow tract velocity-time integrals were significantly decreased in the supine position (*B*, middle panel) and further decreased in the left lateral decubitus position (*A*, left panel). LVOT, left ventricular outflow tract; RVOT, right ventricular outflow tract; VTI, velocity-time integral.

Upon referral, blood pressure was 116/73 mmHg, heart rate was 75 beats/min, and oxygen saturation was 99%. Physical examination revealed a significantly diminished SI and wide splitting of the SII in the left lateral decubitus position, while SI was enhanced and a grade 2/6 systolic ejection murmur appeared in the right lateral decubitus position. Laboratory findings were unremarkable, including N-terminal pro-brain natriuretic peptide level (15 pg/mL) and cardiac troponin T level (0.005 ng/mL). Chest radiography showed a leftward shift of the cardiac silhouette with no tracheal deviation, prominent pulmonary arteries, or interposed lung tissue, resulting in a radiolucent zone between the heart and the diaphragm (*Figure 1A*). Electrocardiography was performed with the patient in various body positions, but lead placement was not altered. The results showed the absence of a transition zone, a QRS axis of 98° (right-axis deviation), and a negative P wave in lead V1, which suggested significant dorsal displacement of the heart in the left lateral decubitus position (*Figure 2A*). However, in the supine position, a transition zone appeared between V5 and V6 with a QRS axis of 111° (*Figure 2B*). Furthermore, the transition zone moved further between V4 and V5, with a QRS axis of 132°, and the polarity of the P wave of V1 became positive in the right lateral decubitus position (*Figure 2C*), suggesting inappropriate anchoring of the heart. Echocardiography revealed that the heart hung far dorsally, with enlargement of the right ventricular (RV) cavity, hyperdynamic motion of the left ventricular (LV) posterior wall (PW), and paradoxical motion of the interventricular septum (IVS) in the left lateral decubitus position (*Figure 3A*; Movies  *S1*, *S2*, and *S3*). In the supine position, however, both the hyperdynamic motion of the PW and the paradoxical IVS motion were alleviated (*Figure 3B*; Movies  *S4*, *S5*, and *S6*). Furthermore, in the right lateral decubitus position, both the cardiac position and morphology completely normalized, and the hyperdynamic motion of the PW, paradoxical motion of the IVS, and RV dilation disappeared (*Figure 3C*; Movies  *S7*, *S8*, and *S9*). The LV eccentricity index (an index of LV deformation; ratio of minor-axis/major-axis LV short-axis dimensions) was significantly changed from 0.66 at left lateral to 0.83 at right lateral decubitus, haemodynamics were also significantly altered during postural changes. Compared with the right lateral decubitus (*Figure 4*, right panel), the velocity-time integral obtained at the RV and LV outflow tracts was significantly decreased in the supine position (middle panel), and further decreased in the left lateral decubitus (left panel). Although advanced echocardiographic techniques, such as speckle-tracking strain analysis and three-dimensional echocardiography, were not employed in this case, conventional Doppler analysis conducted in each position demonstrated that cardiac output varied significantly with body position: 9.5 L/min in the right lateral decubitus position, 6.9 L/min in the supine position, and 6.5 L/min in the left lateral decubitus position.

**Figure 5:**
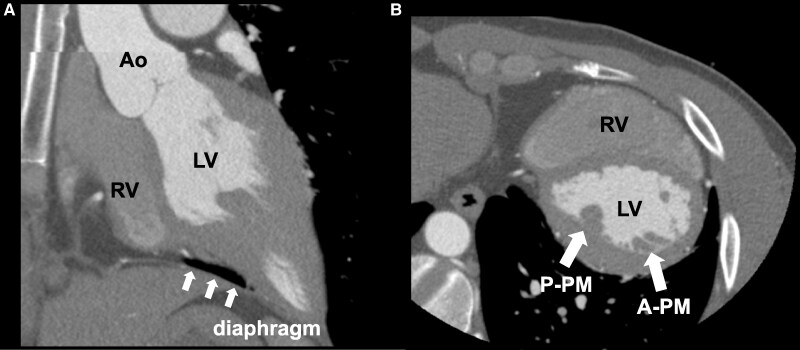
Cardiac multidetector-row computed tomographic images of a 29-year-old man. The right pericardium was partially identifiable; however, this was not confirmed in the left pericardium. Multidetector-row computed tomography shows interposition of the pulmonary tissue between the inferior wall of the LV and the diaphragm (*A*). The anterolateral PM is located posterior to the posteromedial PM because of the significant clockwise rotation of the heart (*B*). A, anterior; Ao, aorta; LV, left ventricle; P, posterior; PM, papillary muscle; RV, right ventricle.

Given a high index of clinical suspicion for a congenital pericardial defect, multidetector-row computed tomography was performed due to its availability at our facility. The imaging clearly demonstrated a complete absence of the left pericardium and interposition of pulmonary tissue between the aorta and the main pulmonary artery (‘lung-tongue sign’, *Figure 1B*) and between the inferior wall of the LV and the diaphragm (*Figure 5A*). The anterolateral papillary muscle (PM) was located more posteriorly than the posteromedial PM due to the significant clockwise rotation of the heart (*Figure 5B*). Computed tomography scans were performed in the supine and left lateral decubitus positions to assess the impact of the pericardial defect on surrounding organs. However, apart from cardiac displacement, no traction of the great vessels, external compression of the lungs, or other anatomical abnormalities were identified that could account for symptoms of chest pain. The integration of multimodal findings led to a final diagnosis of congenital pericardial defects.

Aortic lesions, including aortic dissection, have been reported as potential complications of congenital pericardial defect.^6^ Consequently, regular imaging follow-up is recommended to monitor disease progression. Previous reports have indicated that the long-term prognosis of patients with left-sided pericardial defects does not differ from that of healthy individuals;^7^ therefore, the patient was managed conservatively and was stable after 1 year of follow-up.

## Discussion

A pericardial defect is an extremely rare congenital malformation with an incidence of <1 in 10,000^8^ caused by premature atrophy of the common cardinal vein (Cuvier's canal), resulting in the developmental arrest of the pericardium.^9^ Patients are often asymptomatic and may be detected incidentally during surgery,^10^ autopsy, or radiological studies,^11^ but some cases may present with symptoms including atypical chest pain, palpitation, dyspnoea, dizziness, and rarely, sudden cardiac death.^7^

The pericardium plays an important role in preventing the invasion of pathogenic microorganisms^1^ and avoiding friction with the surrounding structures,^2^ it also prevents unlimited dilation of the heart during an abrupt increase in cardiac preload, thereby exerting a restraining effect on cardiac volume^3,4^ Furthermore, the pericardium anchors the heart in the centre of the thorax,^5^ which might be far more important than previously considered among its functions because when pericardial fixation is impaired by pericardial defects, not only structural but also significant haemodynamic derangements are induced.

When pericardial fixation is impaired, the effects of postural changes on cardiac function are significantly exaggerated. In the left lateral decubitus position, the heart falls dorsally due to gravity, which directly leads to haemodynamic alterations, as those observed in our patient. In this situation, the vertical relationship between the right atrium and the RV is accentuated, which is expected to increase RV diastolic filling due to the increased hydrostatic pressure gradient between the chambers.^12^ As a result of the absence of pericardial restraint, the RV will be overstretched and subsequently enlarges, which may in turn result in compression of the LV via a leftward shift of the IVS and a consequent reduction in cardiac output,^13^ as was observed in this case. Conversely, in the right lateral decubitus position, the heart restores its original anatomical position; thereby, the RV cavity shrinks back to its original size, resulting in an increase in cardiac output due to blunted adverse ventricular interdependence.

Khare *et al*.^14^ previously investigated the effect of body position on electrocardiographic parameters in a cohort of 30 healthy subjects. Their findings demonstrated that the electrical axes of the QRS complex and P wave, QT interval parameters, and the amplitudes of the QRS complex, P wave, and T wave were significantly influenced by four different body positions. However, the magnitude of these changes was relatively small. For example, there was a maximum shift of ∼20° for the QRS axis, 10° for the P wave axis, and about 10% for the QRS voltage. These alterations were considerably less pronounced than those observed in the present case.^14^ Our findings underscore the fact that patients with pericardial defects can exhibit substantial ECG changes in response to postural variations that are not usually observed in healthy individuals. Although shifts in body position can induce slight alterations in cardiac position and volume, even in healthy individuals,^15^ cardiac output is generally reported to remain stable.^16^ This observation suggests that haemodynamic disadvantages in the left lateral decubitus position may be characteristic of pericardial defects, as demonstrated in this case.

We encountered a case of a congenital pericardial defect in which the central anchorage of the pericardium was impaired, resulting in structural, electrophysiological and haemodynamic abnormalities. Through detailed observation of congenital anomalies, haemodynamic insights into the important functions of the pericardium, which were invisible through static observations, were obtained.

## Supplementary Material

ytaf200_Supplementary_Data

## Data Availability

Data is accessible upon reasonable request; however, the authors reserve the right to individually assess and decide upon each request.

## Lead author biography

Manami Shingu graduated in 2020 from Kobe University, Japan. She is currently an internal medicine resident with a major in cardiology in Tamba Medical Center, Japan. Her main areas of interest are heart failure and haemodynamics.

## Notes

**Consent:** The authors confirm that written consent for submission and publication of this case report including images and associated text has been obtained from the patient in line with COPE guidance.

**Funding:** None declared.
